# Detailed analysis of association between common single nucleotide polymorphisms and subclinical atherosclerosis: The Multi-ethnic Study of Atherosclerosis

**DOI:** 10.1016/j.dib.2016.01.048

**Published:** 2016-02-15

**Authors:** Jose D. Vargas, Ani Manichaikul, Xin-Qun Wang, Stephen S. Rich, Jerome I. Rotter, Wendy S. Post, Joseph F. Polak, Matthew J. Budoff, David A. Bluemke

**Affiliations:** a MedStar Health Research Institute, Georgetown University Hospital, Washington, District of Columbia, USA; b Biostatistics Section, Department of Public Health Sciences, University of Virginia, Charlottesville, VA, USA; c Center for Public Health and Genomics, Department of Public Health Sciences, University of Virginia, Charlottesville, VA, USA; d Los Angeles Biomedical Research Institute and Department of Pediatrics, Harbor-UCLA Medical Center, Institute for Translational Genomics and Population Sciences, Torrance, CA, USA; e Division of Cardiology, Johns Hopkins University, Baltimore, MD, USA; f Department of Radiology, Tufts University School of Medicine, Boston, MA, USA; g Los Angeles Biomedical Research Institute, Harbor-UCLA Medical Center, Torrance, CA, USA; h National Institutes of Health, Radiology and Imaging Sciences, Bethesda, MD, USA

**Keywords:** Single nucleotide polymorphism (SNP), Common genetic variant, Subclincal atherosclerosis, Coronary artery calcium (CAC), Carotid intima-media thickness (CIMT)

## Abstract

Previously identified single nucleotide polymorphisms (SNPs) in genome wide association studies (GWAS) of cardiovascular disease (CVD) in participants of mostly European descent were tested for association with subclinical cardiovascular disease (sCVD), coronary artery calcium score (CAC) and carotid intima media thickness (CIMT) in the Multi-Ethnic Study of Atherosclerosis (MESA). The data in this data in brief article correspond to the article Common Genetic Variants and Subclinical Atherosclerosis: The Multi-Ethnic Study of Atherosclerosis [1]. This article includes the demographic information of the participants analyzed in the article as well as graphical displays and data tables of the association of the selected SNPs with CAC and of the meta-analysis across ethnicities of the association of CIMT-c (common carotid), CIMT-I (internal carotid), CAC-d (CAC as dichotomous variable with CAC>0) and CAC-c (CAC as continuous variable, the log of the raw CAC score plus one) and CVD. The data tables corresponding to the 9p21 fine mapping experiment as well as the power calculations referenced in the article are also included.

**Specifications Table**

**Table:** 

Subject area	*Genetics*
More specific subject area	*Cardiovascular genetics*
Type of data	*Tables and figures*
How data was acquired	*Cardiac CT, carotid ultrasound, genotyping*
Data format	*Analyzed*
Experimental factors	*Genetic association studies controlling for CVD risk factors*
Experimental features	*The program R was used to perform genetic association studies*
Data source location	*Multi-Ethnic Study of Atherosclerosis locations across the US*
Data accessibility	*Data is within this article*

**Value of the data**

• Genetic variations play an important role in the atherosclerotic process.
• The data shows novel associations between genetic variations and atherosclerosis.
• The data also shows that previously described genetic associations with atherosclerosis vary considerably depending on ethnicity.
• More research is needed to further elucidate the effect of ethnic-specific genetic variation in cardiovascular disease.

## 1 Data

Previously identified single nucleotide polymorphisms (SNPs) in genome wide association studies (GWAS) of cardiovascular disease (CVD) in participants of mostly European descent were tested for association with subclinical cardiovascular disease (sCVD), coronary artery calcium score (CAC) and carotid intima media thickness (CIMT) in the Multi-Ethnic Study of Atherosclerosis (MESA).

## 2 Experimental design, materials and methods

### 2.1 Study design

The MESA study has been previously described and it was designed to investigate the impact of sCVD and CVD risk factors on the development of clinically overt CVD [2]. Approximately 38% of the recruited participants are Caucasians (EUA), 12% Chinese (CHN), 28% African American (AFA) and 22% Hispanic (HIS). Table 1 describes the demographic characteristics of the participants.

### 2.2 Genotype data

The 66 single nucleotide polymorphisms (SNPs) included in this study (Table 2) were obtained from Affymetrix 6.0 GWAS dataset (MESA and MESA family data) on 8224 consenting MESA participants (2329 EUA, 691 CHN, 2482 AFA, and 2012 HIS) from the National Heart, Lung, and Blood Institute SNP Health Association Resource (SHARe) project. Absent SNPs were imputed using IMPUTE v2.2.2 [3] to the 1000 genomes cosmopolitan Phase 1 v3 as a reference. Genotypes were filtered for SNP level call rate <95% and individual level call rate <95%, and monomorphic SNPs as well as SNPs with heterozygosity >53% were removed. Allele frequencies were calculated separately within each racial/ethnic group, and only those SNPs with minor allele frequencies >0.01 were included in genetic association analyses. We further filtered imputed SNPs based on imputation quality >0.5, using the observed versus expected variance quality metric, and filtered genotyped SNPs for Hardy–Weinberg equilibrium *P*-value≥10^−5^.

### 2.3 SCVD measurement

The imaging outcomes in the present study are coronary artery calcium [CAC, measured as a continuous variable as the raw Agatston CAC score plus one (CAC-c) or as a dichotomous variable (CAC-d) with CAC>0] and carotid artery intima-media thickness [CIMT; internal carotid intima media thickness (CIMT-i), common carotid intima media thickness (CIMT-c)].

CAC was measured by either electron-beam tomography or multi-detector computed tomography, as described previously [4]. All scans were read at the Los Angeles Biomedical Research Institute at Harbor-UCLA Medical Center. Measurements of CAC were adjusted between the different field centers and imaging machines by using a standard calcium phantom of known density, which was scanned with each participant and CAC calculated as described previously [5] and the mean value from two scans used for analysis.

CIMT measurements were performed by B-mode ultrasonography of the right and left, near and far walls, and images were recorded using a Logiq 700 ultrasound device (General Electric Medical Systems, Waukesha, WI). Maximal CIMT-i and CIMT-c was measured as the mean of the maximum values of the near and far wall of the right and left sides at a central ultrasound reading center (Department of Radiology, New England Medical Center, Boston, MA) as described previously [6].

### 2.4 Statistical analyses

**Fig. 1:**
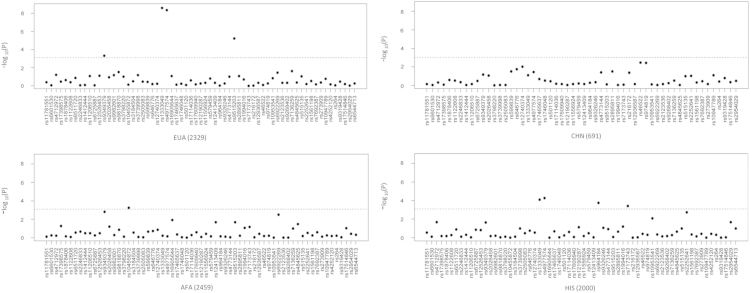
Association of CAC-c (log of the raw CAC score plus one) with CVD and sCVD SNPs by ethnicity. Results from a linear regression assuming an additive model and controlling for age, gender, site of ascertainment, principal components, HDL cholesterol (HDL-C), LDL cholesterol (LDL-C), triglycerides, BMI, hypertension status, diabetes status and tobacco. The dots represent previously identified CVD and sCVD SNPs in prior GWAS as detailed in Table 1. The *y*-axis represents the −log10 of the *p*-value and the dotted line the Bonferroni corrected significance threshold.

**Fig. 2:**
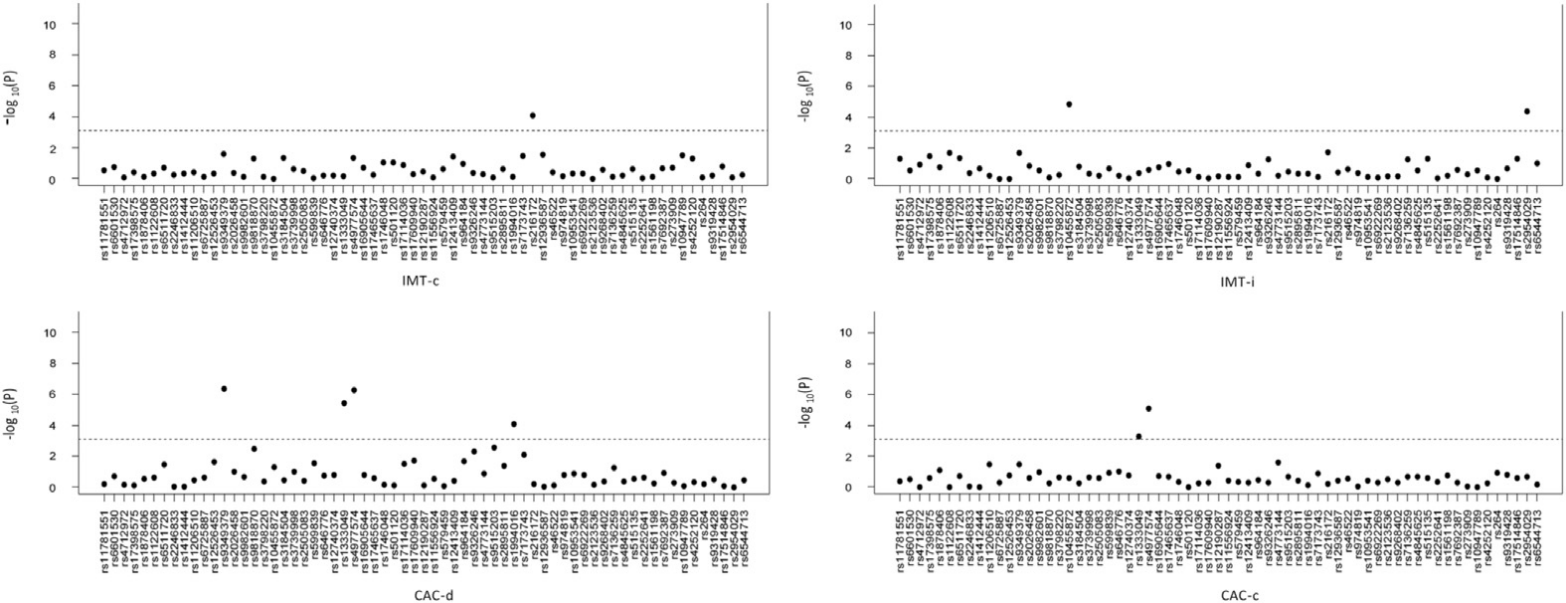
Meta-analysis across ethnicities of the association of CIMT-c (common carotid intima media thickness), CIMT-I (internal carotid intima media thickness), CAC-d (dichotomous variable, CAC>0) and CAC-c (log of the raw CAC score plus one) and CVD and sCVD SNPs. A linear regression assuming an additive model and controlling for age, gender, site of ascertainment, principal components, HDL cholesterol (HDL-C), LDL cholesterol (LDL-C), triglycerides, BMI, hypertension status, diabetes status and tobacco was performed in each ethnic group as described above. The program METAL was used to conduct a fixed effect meta-analysis to combine estimated effects and standard errors from stratified analyses. The dots represent previously identified CVD and sCVD SNPs in prior GWAS as detailed in Table 1. The *y*-axis represents the −log10 of the *p*-value and the dotted line the Bonferroni corrected significance threshold.

Given skewed distributions, the common (CIMT-c) and internal (CIMT-i) IMT values were log normalized. CAC was analyzed as a continuous variable by obtaining the log of the raw CAC score plus one (CAC-c) or as a dichotomous variable (CAC-d) with CAC>0. Analyses were first performed stratified within each racial/ethnic group. For analysis involving EUA and CHN, an unrelated subset of individuals was constructed by selecting at most one individual from each pedigree. For analysis of phenotypes with a substantial familial component, among AFA and HIS, the analysis was performed using a linear mixed-effects model (continuous variables) and by generalized estimating equations (dichotomous variables). Associations between each SNP and each individual phenotype was determined using separate multiple linear regressions (continuous variables) or logistic regressions (dichotomous variables) assuming an additive model. Two models were used to analyze the data. Model 1 accounted for age, sex, site of ascertainment, and principal components. Model 2 included Model 1 plus HDL cholesterol (HDL-C), LDL cholesterol (LDL-C), triglycerides, body mass index (BMI), hypertension status (self-report of physician-diagnosed hypertension along with use of antihypertensive medication or systolic blood pressure of 140 mm Hg or greater and/or diastolic blood pressure of 90 mm Hg or greater), diabetes status (fasting blood glucose was 126 mg/dL or greater or use of diabetes medications), and current smoking use (self-reported current smoking use within the past 30 days). Fixed effect meta-analysis was used to combine results across all four race/ethnic groups, as implemented in METAL. [23] Fig. 1 shows associations of CAC-c by ethnicity. Fig. 2 shows SNP associations with sCVD in a meta-analysis across ethnicities.

Fine mapping of the 9p21 region (100 kb upstream or downstream from SNPs rs1333049, rs4977574, and rs16905644) was performed for each ethnic group by selecting all SNPs on the chromosome 9 imputation set (NCBI Build 37) between positions 21997022–22225503. A total of 3282 SNPs were identified (598, 631, 1256 and 797 SNPs in EUA, CHN, AFA and HIS, respectively). This list of SNPs was supplemented by adding novel SNPs identified by deep sequencing efforts in this region [24], [25]. Given that each ethnicity has its own LD structure, to account for multiple comparisons in each of the race/ethnic-specific analyses, we use an eigen-decomposition to estimate the effective number of independent SNPs in each race/ethnic group [26]. Table 3 shows the association for SNPs in the 9p21 region and CAC-c in EUA and HIS. Table 4 shows the association for SNPs in the 9p21 region and sCVD across ethnicities.

Significance was defined by Bonferroni correction by dividing an alpha of 0.05 by the number of SNPs tested (*p*<7.6×10^−4^ given 66 SNPs tested (0.05/66) for the initial analysis, with greater number of SNPs used for the correction for the fine mapping effort). To assess genetic heterogeneity seen in stratified analyses of the four MESA race/ethnic groups, we used the *I*^2^ heterogeneity metric to quantify the proportion of total variation across studies attributable to heterogeneity rather than chance [27]. Table 5, Table 6 shows power calculations for dichotomous and quantitative traits.

## Sources of funding

MESA and the MESA SHARe project are conducted and supported by contracts N01-HC-95159, N01-HC-95160, N01-HC-95161, N01-HC-95162, N01-HC-95163, N01-HC-95164, N01-HC-95165, N01-HC-95166, N01-HC-95167, N01-HC-95168, N01-HC-95169, UL1-TR-001079 and UL1-TR-000040 from the National Heart, Lung, and Blood Institute (NHLBI, http://www.nhlbi.nih.gov). MESA Family is conducted and supported in collaboration with MESA investigators; support is provided by grants and contracts R01HL071051, R01HL071205, R01HL071250, R01HL071251, R01HL071252, R01HL071258, R01HL071259, M01-RR00425, UL1RR033176, and UL1TR000124. Funding for MESA SHARe genotyping was provided by NHLBI Contract N02‐HL‐6‐4278. The provision of genotyping data was supported in part by the National Center for Advancing Translational Sciences, CTSI grant UL1TR000124, and the National Institute of Diabetes and Digestive and Kidney Disease Diabetes Research Center (DRC) grant DK063491 to the Southern California Diabetes Endocrinology Research Center. This manuscript was approved for submission by the Presentations and Publications Committee.

## Figures and Tables

**Table 1:** Descriptive data for MESA participants whose data was used in this study. Data are presented as *n* (%) for binary measures or median [IQR] for continuous measure.

**Participant characteristics**^a^	**EUA**	**CHN**	**AFA**	**HIS**
No. subjects	2329	691	2482	2012
Women	1212 (52.0)	349 (50.5)	1394 (56.2)	1085 (53.9)
Age, years	63 [54, 71]	63 [54, 71]	60 [53, 68]	60 [53, 68]
BMI, kg/m^2^	27.0 [24.2, 30.3]	23.7 [21.7, 26.0]	29.4 [26.1, 33.8]	28.6 [25.9, 32.0]
Fasting glucose, mg/dL	87 [81, 95]	92 [85, 101]	92 [84, 102]	93 [85, 105]
Hypertension	899 (38.6)	262 (37.9)	1489 (60.0)	830 (41.3)
Diabetes status	128 (5.5)	90 (13.0)	423 (17.0)	369 (18.3)
Lipid medication	422 (18.1)	94 (13.6)	459 (18.5)	333 (16.6)
Current smoking	264 (11.3)	37 (5.4)	480 (19.3)	272 (13.5)
**Lipid levels**^a^				
HDL cholesterol, mg/dL	50 [41, 61]	48 [40, 56]	50 [42, 61]	46 [39, 55]
LDL cholesterol, mg/dL	115 [95, 136]	114 [96, 132]	115 [95, 137]	118 [96, 139]
Total cholesterol, mg/dL	194 [172, 216]	191 [171, 209]	188 [165, 212]	195 [171, 220]
Triglycerides, mg/dL	113 [77, 162]	122 [86, 170]	88 [65, 121]	135 [95, 189]
**Subclinical atherosclerosis**				
Common carotid IMT, mm	0.84 [0.73, 0.97]	0.81 [0.70, 0.92]	0.86 [0.76, 0.99]	0.81 [0.71, 0.93]
Internal carotid IMT, mm	0.89 [0.72, 1.39]	0.73 [0.60, 0.94]	0.91 [0.70, 1.30]	0.83 [0.68, 1.19]
CAC prevalence	1325 (56.9)	356 (51.5)	1063 (43.2)	926 (46.3)
CAC Agatston score^b^	115.7 [23.9, 372.4]	66.4 [21.2, 194.5]	71.7 [17.9, 267.0]	77.3 [20.1, 285.1]

a Sample sizes are reported for participants included in genetic analysis (e.g. participants with all covariates available).

b Agatston score values are reported for participants with CAC>0.

**Table 2:** SNPs previously associated with coronary artery disease (CAD), carotid intima media thickness (CIMT) and coronary artery calcium (CAC).

SNP	Nearest Gene (s)	MAF (risk allele)	*P*-value	GWAS Phenotype	References
rs11781551	ZHX2	0.48 (A)	2.4×10^−11^	CIMT	[7]
rs445925	APOC1	0.11 (G)	1.7×10^−8^	CIMT	[7]
rs6601530	PINX1, SOX7	0.45 (G)	1.7×10^−8^	CIMT	[7]
rs4712972	SLC17A4, SCLC17A1, SLC17A3	0.12 (A)	7.8×10^−8^	CIMT	[7]
rs17398575	PIK3CG	0.25 (T)	2.3×10^−12^	Carotid Plaque	[7]
rs1878406	EDNRA	0.3 (T)	6.9×10^−12^	Carotid Plaque and CAD	[7]
rs1122608	LDLR	0.77(G)	9.7×10^−10^	CAD	[8]
rs6511720	LDLR	0.13 (T)	1.0×10^−7^	Carotid Plaque	[7]
rs2246833	LIPA	0.33 (T)	4.4×10^−8^	CAD	[9]
rs1412444	LIPA	0.33(T)	3.7×10^−8^	CAD	[9]
rs11206510	PCSK9	0.82 (T)	9.10×10^−8^	CAD	[8]
rs6725887	WRD12	0.15(C)	1.2×10^−9^	CAD	[8]
rs12526453	PHACTR1	0.67(C)	1.5×10^−9^	CAD, CAC	[8]
rs9349379	PHACTR1	0.59 (A)	2.65×10^−11^	CAC	[10]
rs2026458	PHACTR1	0.46(T)	1.78×10^−7^	CAC	[10]
rs9982601	MRPS6, SLC5A3, KCNE2	0.15(T)	4.22×10^−10^	CAD	[11]
rs9818870	MRAS	0.16(T)	7.44×10^−13^	CAD, CAC	[12]
rs3798220	LPA	0.02(C)	3.0×10^−11^	CAD, CAC	[13]
rs10455872	LPA	0.30(G)	3.4×10^−15^	CAC	[10]
rs3184504	SH2B3	0.44 (T)	8.6×10^−8^	CAD	[14]
rs3739998	KIAA1462	0.45 (C)	1.27×10^−11^	CAD	[15]
rs2505083	KIAA1462	0.38 (C)	3.87×10^−8^	CAD	[16]
rs599839	SORT1, PSRC1, CELSR2	0.78 (A)	2.89×10^−10^	CAD, LIPID	[8]
rs646776	SORT1, PSRC1, CELSR2	0.81 (T)	7.9×10^−12^	CAD, LIPID, CAC	[17]
rs12740374	SORT1, PSRC1, CELSR2	0.3 (T)	1.8×10^−42^	CAD, LIPID	[18]
rs1333049	CDKN2A, CDKN2B	0.46 (G)	1.35×10^−22^	CAD	[19]
rs4977574	CDKN2A, CDKN2B	0.46 (G)	1.35×10^−22^	CAD, CAC	[19]
rs16905644	CDKN2A, CDKN2B	0.036 (T)	4.1×10^−5^	CAC	[20]
rs17465637	MIA3	0.74 (C)	1.36×10^−8^	CAD	[8]
rs1746048	CXCL12	0.87 (C)	2.93×10^−10^	CAD, CAC	[8]
rs501120	CXCL12	0.83 (A)	7.13×10^–5^	CAD	[21]
rs17114036	PPAP2B	0.91 (A)	3.8×10^−19^	CAD	[8]
rs17609940	ANKS1A	0.75 (G)	1.36×10^−8^	CAD	[8]
rs12190287	TCF21	0.62 (C)	1.07×10^−12^	CAD	[8]
rs11556924	ZC3HC1	0.62 (C)	9.18×10^−18^	CAD	[8]
rs579459	ABO	0.21 (C)	4.08×10^−14^	CAD	[8]
rs12413409	CNNM2, NT5C2, CYP17A1	0.89 (G)	1.03×10^−9^	CAD	[8]
rs964184	APOA5, APOA4, APOA1	0.13 (G)	1.02×10^−17^	CAD	[8]
rs9326246	APOA5, APOA4, APOA1	0.10 ©	2.90×10^–2^	CAD	[21]
rs4773144	COL4A1, COL4A2	0.44 (G)	3.84×10^−9^	CAD	[8]
rs9515203	COL4A1, COL4A2	0.74 (T)	1.13×10^–8^	CAD	[21]
rs2895811	HHIPL1	0.43 (C)	1.14×10^−10^	CAD	[8]
rs1994016	ADAMTS7	0.57 (A)	1.07×10^−12^	CAD	[8]
rs7173743	ADAMTS7	0.58 (T)	6.74×10^–13^	CAD	[21]
rs216172	SMG6, SSR	0.37 (C)	1.15×10^−9^	CAD	[8]
rs12936587	RASD1, SMCR5, PEMT	0.56 (G)	4.45×10^−10^	CAD	[8]
rs46522	UBE2Z, GIP, SNF8	0.53 (T)	1.8×10^−8^	CAD	[8]
rs974819	PDGFD	0.32 (T)	2.41×10^−9^	CAD	[16]
rs10953541	PRKAR2B, HBP1	0.80 (C)	3.12×10^−8^	CAD	[16]
rs6922269	MTHFD1L	0.25 (A)	2.90×10^−8^	CAD	[19]
rs2123536	LINC00954, TTC32, WDR35	0.39 (T)	6.83×10^−11^	CAD	[22]
rs9268402	C6orf10	0.59 (G)	2.77×10^−15^	CAD	[22]
rs7136259	LINC00936, ATP2B1	0.39 (T)	5.68×10^−10^	CAD	[22]
rs4845625	IL6R	0.47 (T)	3.64×10^–10^	CAD	[21]
rs515135	APOB	0.83 (G)	2.56×10^–10^	CAD	[22]
rs2252641	ZEB2, TEX41, BC040861	0.46 (G)	5.30×10^–8^	CAD	[22]
rs1561198	GGCX, RNF181, TMEM150A	0.45 (A)	1.22×10^–10^	CAD	[22]
rs7692387	GUCY1A3, GUCY1B3, TDO2	0.81 (G)	2.65×10^–11^	CAD	[22]
rs273909	SLC22A4, SLC22A5, IRF1	0.14 (G)	9.62×10^–10^	CAD	[22]
rs10947789	KCNK5	0.76 (T)	9.81×10^–9^	CAD	[22]
rs4252120	PLG	0.73 (T)	4.88×10^–10^	CAD	[22]
rs264	LPL	0.86 (G)	2.88×10^–9^	CAD	[22]
rs9319428	FLT1	0.32 (A)	7.32×10^–11^	CAD	[22]
rs17514846	FURIN	0.44 (A)	9.33×10^–11^	CAD	[22]
rs2954029	TRIB1, AK22787	0.55 (A)	4.75×10^–9^	CAD	[22]

**Table 3:** Significant 9p21 SNP associations with CAC-c in EUA and HIS. There were no significant SNPs in AFA and CHN. SNPs were selected 100 kb upstream/downstream from SNPs rs1333049, rs4977574, and rs16905644. A total of 3282 SNPs were identified (598, 631, 1256 and 797 SNPs in EUA, CHN, AFA and HIS, respectively). The Bonferroni corrected *p*-value was determined by dividing 0.05 by the number of SNPs used in which ethnicity.

**SNP**	**Position**	**Beta**	***P*-value**	**MAF**
**EUA**				
rs3218020	21,997,872	0.342	2.09E−07	0.369
rs3217992	22,003,223	0.310	1.58E−06	0.406
rs1063192	22,003,367	0.283	8.43E−06	0.584
rs2069418	22,009,698	0.271	1.80E−05	0.568
rs2069416	22,010,004	0.282	2.50E−05	0.362
rs10,811,641	22,014,137	0.324	5.54E−07	0.385
rs523096	22,019,129	−0.263	2.75E−05	0.423
rs518394	22,019,673	−0.258	3.90E−05	0.422
rs568,447	22,021,615	−0.273	2.06E−05	0.546
rs10738604	22,025,493	0.324	5.75E−07	0.381
rs613312	22,026,594	−0.269	1.94E−05	0.398
rs543830	22,026,639	−0.269	1.94E−05	0.398
rs1591136	22,026,834	0.247	7.82E−05	0.491
rs599452	22,027,402	−0.269	1.94E−05	0.398
rs62560774	22,028,406	−0.267	7.00E−05	0.301
rs679038	22,029,080	−0.269	1.92E−05	0.398
rs10965215	22029445	0.245	8.36E−05	0.493
rs564398	22029547	−0.273	1.46E−05	0.399
rs7865618	22,031,005	0.292	3.39E−06	0.594
rs634537	22,032,152	−0.275	1.29E−05	0.399
rs2157719	22,033,366	0.292	3.45E−06	0.595
rs1008878	22,036,112	0.293	3.31E−06	0.594
rs1556515	22,036,367	0.293	3.30E−06	0.594
rs1333037	22,040,765	0.290	4.56E−06	0.596
rs1412830	22,043,612	−0.268	4.38E−05	0.355
rs1412829	22,043,926	−0.278	1.10E−05	0.400
rs1360589	22,045,317	0.298	2.61E−06	0.596
rs7028268	22,048,414	0.333	2.21E−07	0.407
rs10757265	22,048,859	0.249	7.69E−05	0.491
rs944800	22,050,898	0.298	9.66E−06	0.690
rs944801	22,051,670	0.299	2.34E−06	0.596
rs6475604	22,052,734	0.300	2.26E−06	0.596
rs7030641	22,054,040	0.299	2.41E−06	0.595
rs7039467	22,056,213	0.315	3.49E−06	0.526
rs7853090	22,056,295	0.324	1.39E−06	0.555
rs7866783	22,056,359	0.293	3.63E−06	0.588
rs10757268	22,059,905	0.275	6.16E−05	0.709
rs2095144	22,060,136	0.275	6.20E−05	0.709
rs2383205	22,060,935	0.269	2.81E−05	0.616
rs2184061	22,061,562	0.269	2.83E−05	0.613
rs1537378	22,061,614	0.268	2.96E−05	0.616
rs8181050	22,064,391	0.265	3.42E−05	0.617
rs10811647	22,065,002	0.317	7.24E−07	0.450
rs1333039	22,065,657	0.266	3.09E−05	0.614
rs4977755	22,066,363	0.270	2.58E−05	0.613
rs10965223	22,067,004	0.267	3.25E−05	0.612
rs10965224	22,067,276	0.264	3.52E−05	0.613
rs10811648	22,067,542	0.265	3.39E−05	0.614
rs10811649	22,067,554	0.265	3.26E−05	0.614
rs10811650	22,067,593	0.329	2.26E−07	0.447
rs10811651	22,067,830	0.264	3.55E−05	0.614
rs4977756	22,068,652	0.265	3.30E−05	0.615
rs4451405	22,071,750	0.280	1.90E−05	0.583
rs4645630	22,071,751	0.278	2.21E−05	0.580
rs10757269	22,072,264	0.381	1.33E−09	0.517
rs9632884	22,072,301	0.366	1.05E−08	0.524
rs9632885	22,072,638	0.376	2.00E−09	0.509
rs10757270	22,072,719	0.320	3.68E−07	0.448
rs1831733	22,076,071	0.388	1.98E−09	0.492
rs10757271	22,076,795	0.410	8.83E−11	0.519
rs10811652	22,077,085	0.412	6.79E−11	0.517
rs1412832	22,077,543	0.292	2.11E−05	0.701
rs10116277	22,081,397	0.406	9.13E−11	0.509
rs6475606	22,081,850	0.406	7.71E−11	0.510
rs1547705	22,082,375	0.576	1.31E−05	0.118
rs1333040	22,083,404	0.388	1.12E−09	0.600
rs1537370	22,084,310	0.408	8.66E−11	0.507
rs1,970,112	22,085,598	0.395	3.33E−10	0.500
rs7,857,345	22,087,473	0.311	1.39E−05	0.721
rs10,738,606	22,088,090	0.363	4.56E−09	0.511
rs10,738,607	22,088,094	0.363	4.56E−09	0.511
rs10,757,272	22,088,260	0.360	6.01E−09	0.510
rs10,757,273	22,090,301	0.402	1.89E−09	0.482
rs9,644,859	22,090,521	0.453	3.66E−11	0.455
rs9644860	22,090,603	0.426	1.06E−10	0.485
rs9644862	22,090,936	0.436	1.83E−10	0.449
rs10811653	22,091,069	0.407	9.51E−10	0.479
rs7866503	22,091,924	0.397	4.90E−09	0.466
rs2210538	22,092,257	0.400	7.12E−10	0.494
rs141014318	22,092,924	0.361	8.43E−09	0.499
rs4977757	22,094,330	0.386	1.17E−09	0.501
rs10738608	22,094,796	0.403	1.54E−10	0.520
rs10757274	22,096,055	0.368	3.18E−09	0.512
rs4977574	22,098,574	0.366	3.84E−09	0.511
rs2891168	22,098,619	0.358	7.58E−09	0.511
rs1556516	22,100,176	0.392	3.34E−10	0.528
rs7859727	22,102,165	0.368	3.15E−09	0.515
rs1537372	22,103,183	0.329	2.93E−07	0.451
rs1537373	22,103,341	0.391	3.68E−10	0.528
rs1333042	22,103,813	0.387	5.38E−10	0.531
rs7859362	22,105,927	0.386	8.58E−10	0.535
rs10757275	22,106,225	0.367	5.35E−09	0.519
rs6475609	22,106,271	0.386	8.57E−10	0.535
rs1333043	22,106,731	0.386	8.56E−10	0.535
rs1412834	22,110,131	0.380	1.60E−09	0.536
rs7341786	22,112,241	0.380	1.75E−09	0.538
rs7341791	22,112,427	0.381	1.63E−09	0.538
rs10511701	22,112,599	0.355	1.68E−08	0.527
rs10733376	22,114,469	0.381	1.75E−09	0.532
rs10738609	22,114,495	0.358	1.13E−08	0.520
rs2383206	22,115,026	0.387	8.47E−10	0.531
rs944797	22,115,286	0.387	8.28E−10	0.531
rs1004638	22,115,589	0.381	1.43E−09	0.536
rs2383207	22,115,959	0.382	1.42E−09	0.536
rs1537374	22,116,046	0.381	1.43E−09	0.536
rs1537375	22,116,071	0.354	1.70E−08	0.525
rs1537376	22,116,220	0.387	8.69E−10	0.531
rs1333045	22,119,195	0.349	3.93E−08	0.530
rs10217586	22,121,349	0.327	2.56E−07	0.549
rs10738610	22,123,766	0.355	1.37E−08	0.517
rs1333046	22,124,123	0.357	1.08E−08	0.517
rs7857118	22,124,140	0.380	1.53E−09	0.533
rs10757277	22,124,450	0.378	1.72E−09	0.498
rs10811656	22,124,472	0.394	7.58E−10	0.491
rs10757278	22,124,477	0.378	1.72E−09	0.498
rs1333047	22,124,504	0.405	1.57E−10	0.515
rs10757279	22,124,630	0.379	1.50E−09	0.498
rs4977575	22,124,744	0.405	1.40E−10	0.514
rs1333048	22,125,347	0.350	1.65E−08	0.519
rs1333049	22,125,503	0.371	2.00E−09	0.493
rs1333050	22,125,913	0.332	3.83E−05	0.670
**HIS**				
rs10757270	22,072,719	0.278	5.28E−05	0.422
rs1970112	22,085,598	0.288	3.24E−05	0.473
rs9644860	22,090,603	0.297	5.52E−05	0.442
rs9644862	22,090,936	0.331	7.94E−06	0.482
rs10811653	22,091,069	0.303	4.04E−05	0.434
rs141014318	22,092,924	0.289	5.09E−05	0.399
rs10738608	22,094,796	0.289	4.98E−05	0.542
rs4977574	22,098,574	0.283	5.24E−05	0.412
rs2891168	22,098,619	0.280	5.81E−05	0.416

**Table 4:** Significant SNP associations in the 9p21 in meta-analysis across ethnicities. SNPs were selected 100 kb upstream/downstream from SNPs rs1333049, rs4977574, and rs16905644. A total of 3282 SNPs were identified (598, 631, 1256 and 797 SNPs in EUA, CHN, AFA and HIS, respectively). Chr=Chromosone, Gene=Closest gene, P. Beta=published beta (ethnicity), MAF=Minor allele frequency, H. *P*-value=Heterogeneity *p*-value. *I*^2^=Heterogeneity metric. *P*-values meeting Bonferroni correction are highlighted (the average number of SNPs per ethnicity was used for to derive the Bonferroni corrected *p*-value 0.05/820=6.1E−5).

**SNP**	**Position**	**Beta**	***P*-value**	**EUA**	**CHN**	**AFA**	**HIS**	***I***^**2**^	**H. *P*-value**
**CAC-D**									
rs10757269	22,072,264	0.190	2.38E−05	**+**	**+**	−	**+**	87.4	2.68E−05
rs9632884	22,072,301	−0.194	2.25E−05	−	−	**+**	−	82.3	7.10E−04
rs9632885	22,072,638	−0.186	7.02E−06	−	−	**+**	−	77.9	3.50E−03
rs10757270	22,072,719	0.168	2.93E−05	**+**	**+**	−	**+**	74.9	7.59E−03
rs1831733	22,076,071	0.182	3.05E−05	**+**	**+**	−	**+**	83.1	4.95E−04
rs10757271	22,076,795	0.225	1.46E−06	**+**	**+**	−	**+**	85.1	1.62E−04
rs10811652	22,077,085	0.232	4.10E−07	**+**	**+**	−	**+**	83.1	4.85E−04
rs10116277	22,081,397	−0.247	4.71E−08	−	−	**+**	−	76.5	5.20E−03
rs6475606	22,081,850	−0.247	4.13E−08	−	−	**+**	−	77.6	3.90E−03
rs1333040	22,083,404	−0.176	1.70E−05	−	−	**+**	−	83.2	4.75E−04
rs1537370	22,084,310	−0.226	3.92E−08	−	−	−	−	78.3	3.11E−03
rs1970112	22,085,598	0.220	4.09E−08	**+**	**+**	**+**	**+**	75.6	6.46E−03
rs10738606	22,088,090	0.187	8.17E−06	**+**	**+**	−	**+**	78.6	2.85E−03
rs10738607	22,088,094	0.187	8.17E−06	**+**	**+**	−	**+**	78.6	2.86E−03
rs10757272	22,088,260	−0.190	6.03E−06	−	−	**+**	−	78.5	3.00E−03
rs9644860	22,090,603	−0.176	3.11E−05	−	−	**+**	−	87.5	2.42E−05
rs9644862	22,090,936	0.261	5.58E−09	**+**	**+**	**+**	**+**	70.3	1.77E−02
rs141014318	22,092,924	0.215	6.22E−07	**+**	**+**	−	**+**	72.9	1.13E−02
rs4977757	22,094,330	0.220	5.82E−07	**+**	**+**	**+**	**+**	69.6	1.97E−02
rs10738608	22,094,796	0.255	7.84E−09	**+**	**+**	**+**	**+**	64.9	3.62E−02
rs10757274	22,096,055	0.192	4.26E−06	**+**	**+**	**+**	**+**	77.5	3.94E−03
rs4977574	22,098,574	0.212	5.33E−07	**+**	**+**	−	**+**	71.2	1.52E−02
rs2891168	22,098,619	0.211	4.80E−07	**+**	**+**	**+**	**+**	69.3	2.06E−02
rs1537371	22,099,568	−0.253	2.27E−08	−	−	**+**	−	68.6	2.28E−02
rs1556516	22,100,176	−0.252	2.43E−08	−	−	**+**	−	68.8	2.22E−02
rs7859727	22,102,165	−0.221	1.28E−07	−	−	−	−	71.5	1.46E−02
rs1537372	22,103,183	−0.208	3.87E−06	−	−	**+**	−	65.7	3.29E−02
rs1537373	22,103,341	0.252	2.39E−08	**+**	**+**	−	**+**	68.8	2.21E−02
rs1333042	22,103,813	0.258	1.11E−08	**+**	**+**	−	**+**	68.1	2.42E−02
rs7859362	22,105,927	0.250	4.43E−08	**+**	**+**	−	**+**	65.8	3.24E−02
rs10757275	22,106,225	−0.197	3.69E−06	−	−	**+**	−	76.9	4.70E−03
rs6475609	22,106,271	0.250	4.01E−08	**+**	**+**	−	**+**	65.5	3.37E−02
rs1333043	22,106,731	−0.250	4.10E−08	−	−	**+**	−	65.5	3.37E−02
rs1412834	22,110,131	0.249	5.00E−08	**+**	**+**	−	**+**	65.1	3.50E−02
rs7341786	22,112,241	0.250	5.23E−08	**+**	**+**	−	**+**	64.8	3.65E−02
rs7341791	22,112,427	0.250	5.25E−08	**+**	**+**	−	**+**	64.8	3.63E−02
rs10511701	22,112,599	0.228	8.75E−08	**+**	**+**	**+**	**+**	37.0	1.90E−01
rs10733376	22,114,469	−0.251	5.36E−08	−	−	**+**	−	67.4	2.67E−02
rs10738609	22,114,495	0.201	2.13E−06	**+**	**+**	−	**+**	69.8	1.92E−02
rs1004638	22,115,589	0.251	4.21E−08	**+**	**+**	−	**+**	65.7	3.28E−02
rs2383207	22,115,959	0.252	3.72E−08	**+**	**+**	−	**+**	65.9	3.19E−02
rs1537374	22,116,046	0.252	3.77E−08	**+**	**+**	−	**+**	65.8	3.23E−02
rs1537375	22,116,071	0.240	8.42E−09	**+**	**+**	**+**	**+**	26.9	2.51E−01
rs10738610	22,123,766	0.207	9.77E−07	**+**	**+**	−	**+**	69.3	2.07E−02
rs1333046	22,124,123	−0.179	1.14E−05	−	−	**+**	−	79.4	2.25E−03
rs7857118	22,124,140	0.240	1.53E−07	**+**	**+**	−	**+**	74.8	7.71E−03
rs10757277	22,124,450	0.216	3.84E−07	**+**	**+**	**+**	**+**	71.1	1.55E−02
rs10757278	22,124,477	0.216	3.84E−07	**+**	**+**	**+**	**+**	71.1	1.55E−02
rs1333047	22,124,504	0.264	1.12E−08	**+**	**+**	−	**+**	67.1	2.77E−02
rs10757279	22,124,630	0.220	2.55E−07	**+**	**+**	**+**	**+**	71.8	1.38E−02
rs4977575	22,124,744	0.265	9.46E−09	**+**	**+**	−	**+**	67.4	2.68E−02
rs1333049	22,125,503	−0.189	3.83E−06	−	−	**+**	−	81.2	1.17E−03
**CAC-C**									
rs3218020	21,997,872	−0.173	1.26E−05	−	−	**+**	−	82.4	6.77E−04
rs1063192	22,003,367	−0.185	2.47E−05	−	−	**+**	−	67.1	2.78E−02
rs2069418	22,009,698	−0.197	9.38E−06	−	−	−	−	66.6	2.94E−02
rs10811641	22,014,137	0.160	4.08E−05	**+**	**+**	−	**+**	81.4	1.08E−03
rs523096	22,019,129	−0.185	2.87E−05	−	−	**+**	−	67.0	2.82E−02
rs518394	22,019,673	0.182	3.76E−05	**+**	**+**	−	**+**	65.7	3.29E−02
rs10738604	22,025,493	−0.185	8.13E−06	−	−	**+**	−	81.6	9.87E−04
rs615552	22,026,077	−0.181	4.62E−05	−	−	−	−	61.2	5.17E−02
rs613312	22,026,594	0.185	3.43E−05	**+**	**+**	−	**+**	72.9	1.14E−02
rs543830	22,026,639	−0.185	3.44E−05	−	−	**+**	−	72.9	1.14E−02
rs599452	22,027,402	0.185	3.47E−05	**+**	**+**	−	**+**	72.8	1.15E−02
rs62560774	22,028,406	0.196	5.60E−05	**+**	**+**	−	**+**	68.5	2.30E−02
rs679038	22,029,080	0.183	4.12E−05	**+**	**+**	−	**+**	74.3	8.53E−03
rs564398	22,029,547	−0.182	4.53E−05	−	−	**+**	−	75.4	6.79E−03
rs7865618	22,031,005	−0.205	3.88E−06	−	−	**+**	−	74.2	8.81E−03
rs634537	22,032,152	−0.180	4.85E−05	−	−	**+**	−	76.9	4.61E−03
rs2157719	22,033,366	−0.205	4.30E−06	−	−	**+**	−	73.4	1.04E−02
rs1008878	22,036,112	−0.201	5.03E−06	−	−	**+**	−	75.8	6.10E−03
rs1556515	22,036,367	−0.201	5.24E−06	−	−	**+**	−	75.8	6.15E−03
rs1333037	22,040,765	−0.200	7.52E−06	−	−	**+**	−	73.8	9.52E−03
rs1412829	22,043,926	−0.188	2.69E−05	−	−	**+**	−	75.0	7.46E−03
rs1360589	22,045,317	−0.201	7.78E−06	−	−	**+**	−	75.2	7.04E−03
rs7028268	22,048,414	−0.169	2.40E−05	−	**+**	**+**	−	85.2	1.50E−04
rs944800	22,050,898	0.218	7.26E−06	**+**	**+**	−	**+**	69.5	2.00E−02
rs944801	22,051,670	−0.200	8.51E−06	−	−	**+**	−	75.3	6.88E−03
rs6475604	22,052,734	0.199	8.88E−06	**+**	**+**	−	**+**	75.9	6.04E−03
rs7030641	22,054,040	−0.199	9.32E−06	−	−	**+**	−	75.7	6.28E−03
rs7039467	22,056,213	0.184	4.88E−05	**+**	**+**	**+**	**+**	64.8	3.62E−02
rs7853090	22,056,295	0.214	2.92E−06	**+**	**+**	−	**+**	82.1	7.99E−04
rs7866783	22,056,359	0.190	2.22E−05	**+**	**+**	−	**+**	78.1	3.33E−03
rs10757268	22,059,905	0.206	3.17E−05	**+**	**+**	−	**+**	73.4	1.04E−02
rs2095144	22,060,136	0.206	3.16E−05	**+**	**+**	−	**+**	73.3	1.06E−02
rs2383205	22,060,935	0.184	5.07E−05	**+**	**+**	−	**+**	77.2	4.36E−03
rs1537378	22,061,614	0.184	5.01E−05	**+**	**+**	−	**+**	76.8	4.78E−03
rs8181050	22,064,391	−0.183	5.23E−05	−	−	**+**	−	75.9	6.05E−03
rs10811647	22,065,002	0.161	3.76E−05	**+**	**+**	−	**+**	87.9	1.74E−05
rs10811650	22,067,593	0.169	1.03E−05	**+**	**+**	−	**+**	86.3	7.02E−05
rs10757269	22,072,264	0.248	4.59E−10	**+**	**+**	−	**+**	86.9	4.22E−05
rs9632884	22,072,301	−0.244	1.81E−09	−	−	**+**	−	80.7	1.38E−03
rs9632885	22,072,638	−0.228	7.82E−10	−	−	**+**	−	81.8	9.04E−04
rs10757270	22,072,719	0.206	1.15E−08	**+**	**+**	−	**+**	78.9	2.66E−03
rs1831733	22,076,071	0.236	1.31E−09	**+**	**+**	−	**+**	84.5	2.32E−04
rs10757271	22,076,795	0.280	9.53E−12	**+**	**+**	−	**+**	85.7	1.05E−04
rs10811652	22,077,085	0.282	2.78E−12	**+**	**+**	−	**+**	83.0	5.10E−04
rs1412832	22,077,543	−0.204	1.99E−05	−	−	**+**	−	69.5	2.01E−02
rs10116277	22,081,397	−0.290	3.25E−13	−	−	**+**	−	76.5	5.16E−03
rs6475606	22,081,850	−0.291	2.29E−13	−	−	**+**	−	77.6	3.81E−03
rs1333040	22,083,404	−0.229	4.35E−10	−	−	−	−	83.6	3.87E−04
rs1537370	22,084,310	−0.259	1.86E−12	−	−	−	−	81.6	9.79E−04
rs1970112	22,085,598	0.264	1.81E−13	**+**	**+**	**+**	**+**	75.0	7.40E−03
rs66478960	22,086,826	0.225	6.00E−05	**+**	**?**	**+**	**+**	0.0	4.33E−01
rs7857345	22,087,473	0.229	3.16E−06	**+**	**+**	**+**	**+**	38.1	1.83E−01
rs10738606	22,088,090	0.233	4.84E−10	**+**	**+**	−	**+**	80.4	1.60E−03
rs10738607	22,088,094	0.233	4.84E−10	**+**	**+**	−	**+**	80.4	1.60E−03
rs10757272	22,088,260	−0.233	5.18E−10	−	−	**+**	−	80.0	1.82E−03
rs10757273	22,090,301	−0.201	9.63E−08	−	−	**+**	−	88.7	7.52E−06
rs9644859	22,090,521	−0.217	2.68E−08	−	−	**+**	−	90.5	6.80E−07
rs9644860	22,090,603	−0.221	5.39E−09	−	−	**+**	−	89.6	2.41E−06
rs9644862	22,090,936	0.304	3.19E−14	**+**	**+**	**+**	**+**	70.8	1.65E−02
rs10811653	22,091,069	−0.205	7.62E−08	−	−	**+**	−	90.6	5.20E−07
rs7866503	22,091,924	−0.206	1.08E−07	−	−	**+**	−	87.9	1.68E−05
rs2210538	22,092,257	−0.211	1.87E−08	−	−	**+**	−	89.6	2.40E−06
rs141014318	22,092,924	0.249	8.90E−11	**+**	**+**	**+**	**+**	73.4	1.04E−02
rs4977757	22,094,330	0.251	1.44E−10	**+**	**+**	−	**+**	81.7	9.47E−04
rs10738608	22,094,796	0.289	1.76E−13	**+**	**+**	**+**	**+**	70.8	1.65E−02
rs4977574	22,098,574	0.252	2.55E−11	**+**	**+**	**+**	**+**	73.3	1.05E−02
rs2891168	22,098,619	0.244	7.19E−11	**+**	**+**	**+**	**+**	74.8	7.69E−03
rs1537371	22,099,568	−0.284	1.01E−12	−	−	−	−	73.1	1.09E−02
rs1556516	22,100,176	−0.283	1.17E−12	−	−	−	−	73.5	1.01E−02
rs7859727	22,102,165	−0.245	4.40E−11	−	−	−	−	77.9	3.55E−03
rs1537372	22,103,183	−0.248	6.93E−10	−	−	**+**	−	65.5	3.37E−02
rs1537373	22,103,341	0.283	1.22E−12	**+**	**+**	**+**	**+**	72.9	1.14E−02
rs1333042	22,103,813	0.283	1.10E−12	**+**	**+**	**+**	**+**	72.7	1.18E−02
rs7859362	22,105,927	0.276	6.92E−12	**+**	**+**	**+**	**+**	70.5	1.72E−02
rs10757275	22,106,225	−0.235	5.68E−10	−	−	**+**	−	78.4	3.08E−03
rs6475609	22,106,271	0.277	5.91E−12	**+**	**+**	**+**	**+**	69.9	1.88E−02
rs1333043	22,106,731	−0.277	6.27E−12	−	−	−	−	69.9	1.89E−02
rs1412834	22,110,131	0.274	1.09E−11	**+**	**+**	**+**	**+**	69.4	2.04E−02
rs7341786	22,112,241	0.276	1.03E−11	**+**	**+**	**+**	**+**	68.2	2.39E−02
rs7341791	22,112,427	0.276	9.76E−12	**+**	**+**	**+**	**+**	68.4	2.33E−02
rs10511701	22,112,599	0.255	2.01E−11	**+**	**+**	**+**	**+**	56.9	7.34E−02
rs10733376	22,114,469	−0.279	7.26E−12	−	−	−	−	69.7	1.93E−02
rs10738609	22,114,495	0.235	5.10E−10	**+**	**+**	**+**	**+**	72.2	1.30E−02
rs2383206	22,115,026	0.185	3.13E−07	**+**	**+**	−	**+**	89.4	2.97E−06
rs944797	22,115,286	0.186	2.69E−07	**+**	**+**	−	**+**	89.5	2.92E−06
rs1004638	22,115,589	0.275	9.00E−12	**+**	**+**	**+**	**+**	69.8	1.91E−02
rs2383207	22,115,959	0.275	8.92E−12	**+**	**+**	**+**	**+**	69.8	1.92E−02
rs1537374	22,116,046	0.276	8.19E−12	**+**	**+**	**+**	**+**	69.8	1.91E−02
rs1537375	22,116,071	0.266	8.73E−13	**+**	**+**	**+**	**+**	43.6	1.50E−01
rs1537376	22,116,220	0.185	3.29E−07	**+**	**+**	−	**+**	89.4	2.96E−06
rs1333045	22,119,195	0.182	2.89E−07	**+**	**+**	−	**+**	86.4	6.24E−05
rs10217586	22,121,349	0.172	1.21E−06	**+**	**+**	−	**+**	84.9	1.77E−04
rs10738610	22,123,766	0.235	4.94E−10	**+**	**+**	**+**	**+**	72.4	1.25E−02
rs1333046	22,124,123	−0.210	9.85E−09	−	−	**+**	−	82.0	8.27E−04
rs7857118	22,124,140	0.270	2.30E−11	**+**	**+**	−	**+**	77.5	4.02E−03
rs10757277	22,124,450	0.248	6.77E−11	**+**	**+**	**+**	**+**	75.4	6.68E−03
rs10811656	22,124,472	−0.198	6.29E−08	−	−	**+**	−	88.6	8.51E−06
rs10757278	22,124,477	0.248	6.78E−11	**+**	**+**	**+**	**+**	75.4	6.67E−03
rs10757277	22,124,450	0.248	6.77E−11	**+**	**+**	**+**	**+**	75.4	6.68E−03
rs10811656	22,124,472	−0.198	6.29E−08	−	−	**+**	−	88.6	8.51E−06
rs10757278	22,124,477	0.248	6.78E−11	**+**	**+**	**+**	**+**	75.4	6.67E−03
rs1333047	22,124,504	0.296	3.23E−13	**+**	**+**	**+**	**+**	70.4	1.76E−02
rs10757279	22,124,630	0.250	4.65E−11	**+**	**+**	**+**	**+**	75.6	6.46E−03
rs4977575	22,124,744	0.297	2.65E−13	**+**	**+**	**+**	**+**	70.5	1.72E−02
rs1333048	22,125,347	0.186	2.32E−07	**+**	**+**	−	**+**	85.0	1.66E−04
rs1333049	22,125,503	−0.218	2.42E−09	−	−	**+**	−	84.6	2.18E−04
rs1333050	22,125,913	−0.199	8.65E−06	−	−	**+**	−	69.3	2.05E−02

**Table 5:** Power to detect a genetic additive effect assuming a type I error rate of <7.6×10^−4^ given 66 SNPs tested (0.05/66) for a dichotomous trait with a prevalence of 50% as a function of minor allele frequency (MAF) and genetic relative risk (GRR). The prevalence of CAC in MESA varies according to age, gender and ethnicity and could be either slightly above or below 50% depending on these factors. The samples sizes used in the power calculation encompass those of the different ethnic groups in MESA (European Americans 2329, African Americans 2482, Hispanic Americans 2012 and Chinese Americans 691).

**MAF**	**GRR**	**Power (*n*=800)**	**Power (*n*=1700)**	**Power (*n*=2600)**
0.06	1.1	0.0031	0.0058	0.0093
	1.2	0.0104	0.0296	0.0586
	1.3	0.0275	0.0943	0.1950
0.11	1.1	0.0048	0.0109	0.0191
	1.2	0.0220	0.0731	0.1514
	1.3	0.00675	0.2430	0.4622
0.16	1.1	0.0066	0.0165	0.0307
	1.2	0.0359	0.1267	0.2592
	1.3	0.1172	0.4009	0.6759
0.21	1.1	0.0083	0.02223	0.0431
	1.2	0.0508	0.1830	0.3627
	1.3	0.1700	0.5365	0.8105

**Table 6:** Power to detect a genetic additive effect assuming a type I error rate of 7.6×10^−4^ given 66 SNPs tested (0.05/66) for a quantitative trait with a population standard deviation of 0.11 as a function of SNP effect size (beta) and minor allele frequency (MAF). The estimation of standard deviation as well as SNP effect size are based on published IMT and genetic association data. The samples sizes used in the power calculation encompass those of the different ethnic groups in MESA (European Americans 2329, African Americans 2482, Hispanic Americans 2012 and Chinese Americans 691).

**MAF**	**Beta**	**Power (*n*=800)**	**Power (*n*=1700)**	**Power (*n*=2600)**
0.06	0.0100	0.0062	0.0175	0.0351
	0.0160	0.0236	0.0881	0.1907
	0.0220	0.0714	0.2759	0.5244
0.11	0.0100	0.0129	0.0438	0.0941
	0.0160	0.0611	0.2385	0.4672
	0.0220	0.1950	0.6135	0.8757
0.16	0.0100	0.0210	0.0773	0.1678
	0.0160	0.1090	0.3998	0.6855
	0.0220	0.3347	0.8210	0.9734
0.21	0.0100	0.0297	0.1137	0.2433
	0.0160	0.1602	0.5370	0.8191
	0.0220	0.4608	0.9190	0.9942
